# Optimization of the cardiac delirium index by including age, decrease in butyrylcholinesterase actitivity, preoperative HbA1c, and postoperative hemoglobin levels: results of a secondary analysis of a prospective observational study

**DOI:** 10.3389/fcvm.2024.1459268

**Published:** 2024-12-06

**Authors:** Thomas S. Zajonz, Fabian Edinger, Melanie Markmann, Katrin Gräb, Michael Sander, Christian Kunzemann, Christian Koch, Emmanuel Schneck

**Affiliations:** Department of Anaesthesiology, Operative Intensive Care Medicine and Pain Therapy, University Hospital of Giessen, Justus-Liebig University Giessen, Giessen, Germany

**Keywords:** biomarker, postoperative delirium, cardiac surgery, cardiac artery bypass graft surgery, cholinergic system

## Abstract

**Introduction:**

Postoperative delirium (POD) after cardiac surgery significantly affects the perioperative morbidity and mortality. Butyrylcholinesterase (BChE) is an enzyme primarily produced in the liver, which plays a crucial role in the hydrolysis of acetylcholine outside of neuronal synapses, referred to as extraneuronal hydrolysis. The integration of BChE activity into the cardiac delirium (CARDEL) index might increase its predictive power for identifying POD after cardiac surgery. Therefore, the primary aim of this study was to assess the applicability of the CARDEL index and determine whether integrating the BChE activity enables optimization of the predictive model.

**Methods:**

This secondary analysis of a prospective observational study included patients undergoing elective coronary artery bypass graft surgery. BChE activity is expressed in units per liter (U/L), while the BChE drop refers to the percentage decrease in BChE activity from pre- to postoperative levels. POD risk factors were identified using multivariate regression analysis. The predictive power of the CARDEL index and an optimized model including BChE was calculated with receiver operating characteristic (ROC) analysis.

**Results:**

Of 93 included patients, 20 (21.5%) developed POD. Elevated preoperative HbA1c [OR 2.5 (1.2–4.8), *p* = 0.01], a decrease in BChE activity [%, OR 1.1 (1.0–1.2), *p* = 0.04], age [1 (0.94–1.1), *p* = 0.55], and a postoperative hemoglobin change [OR 0.86 (0.78–0.96), *p* < 0.001] were identified as independent risk factors for POD. While the CARDEL index showed a moderate prediction of POD [AUCROC of 0.74 (0.60–0.87)], the optimization including BChE resulted in a significant prognostic improvement: AUCROC of 0.84 (0.72–0.94, *p* < 0.001).

**Conclusion:**

Despite the small size of this derivation cohort, this study identified elevated HbA1c as the strongest risk factor for the development of POD, followed by a decrease in BChE activity, postoperative anemia, and age, respectively. By including these parameters to the CARDEL index, its predictive power for the identification of POD significantly improved in this derivation cohort. Moving forward, integrating these findings into clinical practice could enhance early risk stratification and targeted intervention for patients at high risk of POD. Therefore, further research should evaluate these results in a larger, external cohort.

## Introduction

Delirium represents an acute and fluctuating disturbance of cerebral function associated with changes in consciousness and attention (1, 2). It is classified by various features, such as predominant motor activity (hypoactive, hyperactive, or mixed) or etiology (hypoxia, sepsis, sedative exposure, or metabolic dysfunction). Different phenotypes can be subdivided, with hyper- and hypoactive symptoms or their combinations, while some patients show an acute change in cognition that does not meet the full criteria for delirium and is described as subsyndromal delirium (3).

Particularly after cardiac surgery, postoperative delirium (POD) presents a common complication that severely impairs the patients' well-being and significantly increases their perioperative morbidity and mortality as well as health care costs (4, 5). The incidence of POD in cardiac surgical patients varies from 16% to nearly 60%, depending on age, pre-existing cerebrovascular disease and cognitive function, procedural factors (e.g., extent of surgical trauma, duration of extracorporeal circulation), and anesthetic management (6–8). Although numerous underlying pathomechanisms of POD following cardiac surgery have been identified, most are not yet fully understood.

In addition to the neuroinflammatory hypothesis, the neurotransmitter hypothesis represents one of the most widely accepted pathophysiological models for cognitive disturbances. It centers on an imbalance of neurotransmitters such as dopamine, acetylcholine, and serotonin in the brain. Specifically, disturbances in butyrylcholinesterase (BChE) activity can lead to a deficiency of acetylcholine in humans, as BChE plays a key role in regulating acetylcholine levels by hydrolyzing it in non-neuronal tissues (9, 10). While acetylcholinesterase primarily functions at neuronal synapses, BChE contributes to the breakdown of acetylcholine in peripheral tissues and plays a modulatory role in overall cholinergic signaling (11, 12). It is primarily expressed in the liver. In the context of POD, emerging evidence suggests that alterations in BChE activity may influence cholinergic dysregulation, contributing to the cognitive disturbances seen in delirium or other dementia-related diseases (13–16). Alongside other studies, own data showed alterations in the BChE activity, which were predictive of POD in cardiac surgical patients (17–19). However, although a recent study (*n* = 237) confirmed a decrease in BChE activity after cardiac surgery, it was not an independent risk factor for POD development (20). The authors of this study assumed that the BChE activity resembles a surrogate parameter for the patient's overall condition rather than a specific diagnostic tool for POD. Thus, it might be rational to combine the BChE activity with a clinical tool such as the CARDEL index for distinguishing POD after cardiac surgery.

While biomarkers of neuroinflammation are promising, they are often compromised by the immunomodulatory effects of cardiopulmonary bypass (CPB), and the use of clinical scores is limited due to necessary postoperative analgesia and sedation (3, 4, 21–23). This dilemma could be resolved by combining biomarkers and clinical scoring tools that consider relevant patient characteristics (e.g., age), which might optimize risk stratification for POD, even in sedated, uncooperative, and ventilated patients. In this context, Kotfis et al. stated that a lower preoperative platelet-to-white-blood-cell ratio (PWR) correlated well with POD after cardiac surgery (*n* = 968). Consequently, they introduced the cardiac delirium index (CARDEL index) including the PWR, age, and HbA1c and demonstrated a sufficient predictive power (AUC of 0.742) for the occurrence of POD following cardiac surgery (21). However, the CARDEL index has not yet been reevaluated and it has not yet been analyzed in combination with the drop of BChE activity.

The primary aim of this study is to assess the applicability of the CARDEL index in a cohort of patients undergoing elective on-pump cardiac surgery. Furthermore, the secondary aim is to determine whether integrating the BChE activity enables optimization of the predictive model.

## Methods

### Study design

This study presents a secondary analysis of an observational study including 100 cardiac surgical patients at the University Hospital of Giessen and was registered in the German Clinical Trials Register (trial registration: DRKS00010959). The aim of the primary study was to evaluate whether pre- and perioperative changes in blood acetylcholinesterase and BChE activity were associated with POD development in patients undergoing isolated elective coronary artery bypass graft (CABG) surgery (17). Local ethics committee approval was obtained (Justus Liebig University Giessen, Giessen, Germany; approval number AZ: 30/16). Written informed consent was obtained from all patients. The study was conducted according to the principles of the Declaration of Helsinki (24). The methods and results are presented according to the Strengthening the Reporting of Observational Studies in Epidemiology (STROBE) guidelines (25).

### Subjects

Patients were recruited between September 2016 and January 2020 and included those undergoing elective coronary artery bypass graft (CABG) surgery and postoperative intensive care unit (ICU) treatment. Overall, 100 patients were originally included in the study. Three patients were excluded due to pre-existing delirium, while four declined to participate; therefore, 93 patients were included for analysis. Inclusion criteria included age ≥18 years, elective on-pump CABG surgery, and the ability to communicate in German or English. Patients were excluded in case of missing consent, denial of participation, pregnancy, preoperative atrial fibrillation, severe bradycardia (<60 bpm; types: sinus bradycardia, atrial fibrillation with low frequency, nodal rhythm, and second- or third-degree atrioventricular block), acute infection before surgery, pre-existing autoimmune disease, immunomodulatory medication, left ventricular ejection fraction <30%, and renal insufficiency (Kidney Disease Improving Global Outcome score >2). Further exclusion criteria were pseudocholinesterase deficiency, preoperative delirium [screened using the Confusion Assessment Method for the Intensive Care Unit [CAM-ICU] and/or Intensive Care Delirium Screening Checklist [ICDSC]], cognitive dysfunction (e.g., history of schizophrenia, other severe psychiatric conditions, or dementia with inability to adequately answer the CAM-ICU or ICDSC), and recent or persistent neurological impairment (e.g., acute cerebral infarction, intracranial bleeding, or acute meningitis in the last three months prior to study inclusion leading to inability to answer the CAM-ICU or ICDSC).

### Identification of delirium

All patients received a standardized perioperative anesthetic and hemodynamic treatment, which is described in detail in the primary study publication (17). Prior to surgery, patients were asked to perform the ICDSC and CAM-ICU tests to achieve a baseline assessment. Postoperatively, POD development was monitored for seven days by applying the ICDSC and CAM-ICU daily during the observational period. One investigator performed all examinations under the supervision of the principal investigator and the attending intensive care physicians to exclude investigator bias. Sedation status was examined using the Richmond Agitation-Sedation Scale: Patients with a score of ≤−2 were excluded from testing and reevaluated after four hours. Patients with a positive result with either score were considered to have POD [as shown in the primary study (17)]. In the daily ICU routine, CAM-ICU and RASS were used for detecting POD.

### BChE measurements

Preoperative baseline analysis of BChE activity was performed after the clinical examination prior to surgery, while postoperative measurements included one analysis on each consecutive day for seven days after surgery. Whole blood samples (10 µl) were drawn from a peripheral intravenous catheter and analyzed using the ChE Check Mobile System (Securetec Detektions-Systeme AG, Neubiberg, Germany). The BChE activity was analyzed by following the procedural steps according to the manufacturer's instructions [for a detailed description, see (26, 27)]. The enzyme kinetics of BChE was determined photometrically with a modified spectrophotometric Ellman assay (26). BChE activities are measured at 470 nm and are normalized to 37°C. The results of BChE activity measurements are expressed in units per liter (U/L), while the BChE drop refers to the percentage decrease in BChE activity from pre- to postoperative levels.

All other laboratory data originate from clinical routine laboratory blood sampling.

### Statistical analysis

Descriptive analysis included the calculation of median and interquartile ranges. Any operative or postoperative ratios refer to the preoperative values. Numerical values were tested for normal distribution using the Shapiro test, and tests for statistically significant differences between the POD- and POD+ groups were applied accordingly with the Student's *t*-test or Wilcoxon test. Since red blood cell concentrates (RBC) can influence plasma BChE activity, their correlation with BChE levels was analyzed using Pearson correlation analysis (28).

The CARDEL index provided by Kotfis et al. was used with the beta coefficients given as well as adapted by retrieving beta coefficients for the present data set (21). It included the following items in its formula:

CARDELIndex=0.108×Age+0.341×HBA1C−0.049×PWR

The CARDEL index adapted to our data was used for graphical display and comparisons.

The predictive power of the CARDEL index was calculated by receiver operating characteristic (ROC) and quantified with the area under the curve (AUCROC). Confidence intervals for the AUC were calculated with the “bootstrap” method and *n* = 2000. The results of the BChE measurements were added to the variable set for the CARDEL index, and the AUCROC was calculated accordingly.

Logistic regression was performed, and statistically significant variables from this univariate analysis were additionally included to compose other multivariable models to identify influencing parameters for optimizing the CARDEL index. Different models were calculated, and the statistical significance of the resulting models was determined by comparisons with the null model. Variables were included according to the parameter's significance and ease of measurement.

Subsequently, indices were calculated based on the different models according to the respective resulting beta coefficients. The statistical significance of the difference in indices between the POD- and POD+ groups was determined with a *t*-test. Statistical significance was assumed with *p*-values below 0.05.

Due to the study design, it was illogical to perform a sample size calculation in advance, as this was a secondary analysis of a prospective study after patient recruitment had been completed. Thus, the number of patients could not be adjusted.

All analyses were performed using R Statistical Software (version 4.3.2, 2023-10-31, R Core Team 21, https://www.r-project.org/).

## Results

### Baseline characteristics

Table 1 shows the patient characteristics [as previously shown in the primary study (17)]. An assessment regarding neurological and psychiatric diseases was performed; it revealed that eight patients (8.6%) had a history of cerebral infarction without persistent neurological impairment, and five patients had a history of psychiatric disorder (5.4% depression, post-traumatic stress disorder, or obsessive-compulsive disorder). No patient suffered from dementia and no drug or medication abuse was reported, while 31 patients (33.3%) reported regular alcohol consumption. As demonstrated in the original publication, liver enzymes did not differ between patients with and without POD (17). No patient suffered from a severe liver disease (≥Child A). No patient died during the study period.

**Table 1:** Baseline characteristics of POD+ and POD− patient groups.

Characteristic	POD+ (*n*=20)	POD– (*n*=73)	*p*-value
Age (years)	71.5 (63.8–76.3)	64 (56–71)	0.003
Female sex	3 (15.0)	9 (12.3)	0.717
Caucasian (%)	20 (100)	73 (100)	n.a.
Body mass index (kg/m^2^)	28.9 (25.7–32.4)	29.4 (27–32.1)	0.581
Atrial fibrillation	3 (15)	19 (26)	0.385
COPD	5 (25)	6 (8.2)	0.054
DM II	13 (65)	22 (30.1)	0.008
EuroSCORE	1.4 (1.2–1.8)	0.9 (0.7–1.2)	<0.001
ASA–PSCS			0.30
II	0	7 (9.6)	–
III	19 (95.0)	57 (78.1)	–
IV	1 (5.0)	9 (12.3) ()	–
Duration of ventilation (h)	20.8 (17.0–24.2)	12.3 (9.4–16.6)	<0.001
LOS-ICU (d)	2.9 (5.2–7.2)	1.1 (0.9–2.0)	<0.001
LOS-Hospital (d)	11.0 (9.3–13.8)	10.0 (8.0–12.0)	0.125

Numerical variables are accompanied by medians and IQRs, with categorical data presented as percentages. ASA-PSCS, American Society of Anesthesiologists—physical status classification system; COPD, chronic obstructive pulmonary disease; DM II, diabetes mellitus type II; IQR, interquartile range; POD = postoperative delirium.

### Onset of delirium

In summary, 20 patients (21.5%) developed POD, of whom 94.6% showed consistent CAM-ICU and ICDSC results. In five patients (5.4%), the CAM-ICU and ICDSC results differed, leading to a clinical diagnosis based on the ICD-10 classification. As shown in the primary paper, most patients developed POD within the first 24 postoperative hours.

### BChE activity

**Figure 1:**
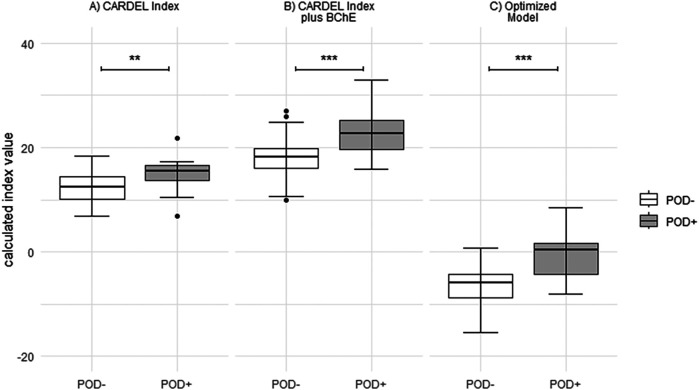
Boxplot demonstrating the differences in indices between both study groups calculated as **(A)** CARDEL index, **(B)** CARDEL index with drop in BChE activity included, and **(C)** index calculated from the optimized model. Box and whisker plots indicate median, interquartile range (box), minimum and maximum (whiskers). ***t-test *p*-value <0.001, **t-test *p*-value <0.01.

While preoperative BChE activity did not differ between patients with and without POD, it demonstrated significant differences at the postoperative time point (POD+: BChE activity preoperative 3,158.5 [3,018.3–3,512.1] U/L vs. BChE activity postoperative 2,189.3 [1,917.5–2,364.9] U/L; POD-: BChE activity preoperative 3,180.9 [2,822.9–3,472.1] U/L vs. BChE activity postoperative 2,312.3 [2,093.5–2,725.3] U/L; *p*-value <0.001 for intergroup difference at the postoperative time point; Figure 1).

BChE activity (U/L) showed a negative correlation with the amount of RBC administered, although the data exhibited high variability (correlation coefficient: −0.26, adjusted *R*^2^: 0.06, *p* = 0.01). In summary, 10 out of 20 (50%) patients with POD received RBCs, while 15 out of 73 (20.5%) patients without POD were treated with them. Patients with POD received more RBC compared to patients without POD [median of absolute amount RBC; POD+: 450.0 [0–600] ml; POD−: 0 [0] ml].

### Assessment of the CARDEL index

Overall, the original CARDEL index and the CARDEL index adapted to our data differed significantly between the study groups (original: POD- 8.75 [8–9.5] vs. POD+ 9.9 [9.2–10.2], *p* < 0.003; adapted: POD- 12.4 [10.1–14.5] vs. POD+ 15.6 [13.6–16.6], *p* < 0.01; in 14 of the POD- patients and two patients in the POD+ group, the CARDEL index was not calculable due to missing HbA1c values; Table 2, Figure 1). The CARDEL index (both original and adapted) had moderate predictive power for predicting POD [AUCROC original 0.74 [0.61–0.86] and adapted 0.74 [0.60–0.87]; Figure 1].

**Table 2:** Results of univariate analysis.

	Odds ratio (unadjusted)	Confidence interval	*p*-value
Age (years)	1.08	1.02–1.15	<0.01
Hb1Ac (%)	1.53	1.03–2.28	<0.05
BChE activity (U/L Hb)			
Preoperative BChE	1.000	0.999–1.001	0.77
Postoperative BChE	0.998	0.997–1.00	<0.05
Pre- to postoperative BChE drop (%)	1.12	1.04–1.19	<0.01
Postoperative BChE proportion (%)	0.90	0.84–0.96	<0.01
Platelets (giga/L)			
Preoperative platelet count	0.99	0.99–1.00	0.12
Intraoperative platelet count	0.99	0.98–1.00	<0.05
Postoperative platelet count	0.99	0.97–1.00	<0.05
Ppostoperative proportion of platelets (%)	1.00	0.98–1.02	0.81
Hemoglobin (g/dl)			
Preoperative hemoglobin	1.02	0.98–1.05	0.34
Intraoperative hemoglobin	0.95	0.92–0.99	<0.05
Postoperative hemoglobin	0.95	0.91–0.99	<0.05
Postoperative proportion of hemoglobin (%)	0.90	0.84–0.96	<0.01
Hematocrit (Vol%)			
Preoperative hematocrit	1.09	0.95–1.25	0.22
Intraoperative hematocrit	0.91	0.80–1.05	0.20
Postoperative hematocrit	0.87	0.76–1.00	< 0.05
Postoperative proportion of hematocrit (%)	0.91	0.85–0.97	< 0.01
Erythrocytes count (tera/L)			
Preoperative erythrocyte count	1.31	0.47–3.66	0.60
Intraoperative erythrocyte count	0.45	0.15–1.32	0.15
Postoperative erythrocyte count	0.28	0.09–0.89	<0.05
Postoperative proportion of erythrocytes (%)	0.91	0.85–0.97	<0.01
WBC count (giga/L)			
Preoperative WBC count	1.07	0.88–1.31	0.51
Intraoperative WBC count	0.96	0.86–1.06	0.39
Postoperative WBC count	0.91	0.79–1.05	0.19
Postoperative proportion of WBC (%)	0.99	0.98–1.00	<0.05
Platelet-to-WBC ratio			
Preoperative Platelet-to-WBC ratio	0.96	0.92–1.01	0.15
Intraoperative Platelet-to-WBC ratio	0.97	0.89–1.05	0.44
Postoperative Platelet-to-WBC ratio	0.97	0.94–1.01	0.13
Pre- to postoperative change of platelet-to-WBC ratio (%)	1.005	0.99–1.02	0.56

BChE, butyrylcholinesterase; WBC, white blood cells.

### Optimization of the CARDEL index

Since the predictive performance of the CARDEL index was only moderate, we included the decrease in BChE activity, which improved the index's performance [AUC = 0.80 [0.67–0.91], POD- 18.2 [16.0–19.9] vs. POD+ 22.7 [19.7–25.3], *p* < 0.001].

**Figure 2:**
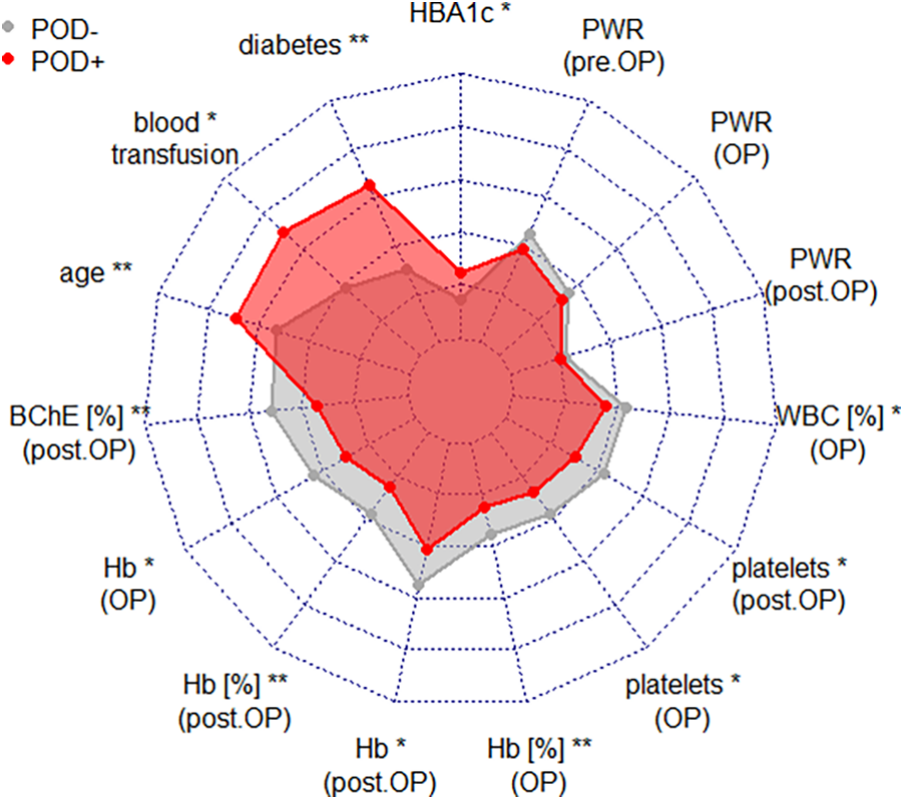
Spiderweb plot demonstrating the comparison of parameters resulting from univariate logistic regression analysis and those used in the CARDEL index calculation. *p < 0.05, **p < 0.01. Hb, hemoglobin; WBC, white blood cells; BChE, butyrylcholinesterase; HbA1c, glycated hemoglobin; POD, postoperative delirium; PWR, platelet-to-white-blood-cell ratio.

Univariate analysis was used to identify additional risk factors for POD development (Figure 2, Table 2). However, with our data set, the PWR could not be confirmed as an associated factor for the occurrence of POD.

Instead, any decrease in red-blood-cell-related parameters appeared to impact the POD risk (Figure 2, Table 2). Consequently, we included these parameters in a multivariable logistic regression analysis and excluded the PWR. The most potent parameters regarding POD prediction were age, HbA1c [OR 2.5 (1.2–4.8), *p* = 0.01], relative decrease in BChE activity [in%, OR 1.1 (1.0–1.2), *p* = 0.04], and the postoperative proportion of hemoglobin [OR 0.86 (0.78–0.96), *p* < 0.001], and these were consequently included in the optimized model.

The formula for calculating an index according to this optimized model is:

Idx=0.11×age[y]+0.29×preHbA1c+0.26×dropinBChEactivity[%]−0.28×proportionofHbpostOP[%]

**Figure 3:**
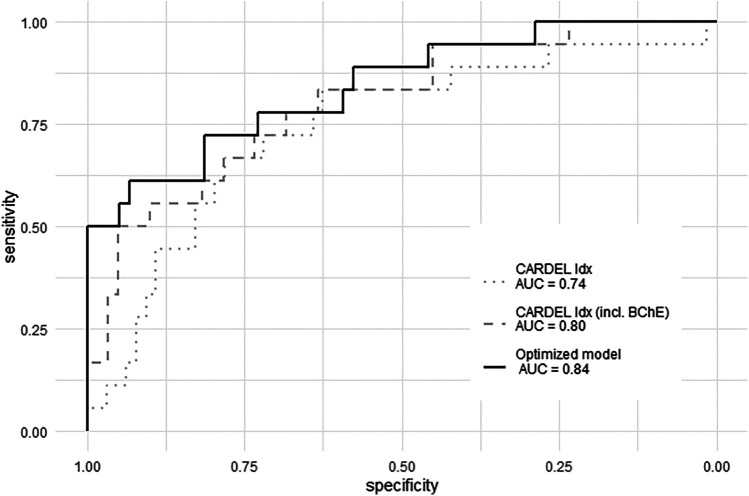
ROC for the CARDEL index (dotted grey line), the extended CARDEL index (dotted dark grey line), and the index according *to the optimized model (solid black line)*. BChE, butyrylcholinesterase; CARDEL, cardiac delirium index; ROC, receiving operator curve.

Table 3 shows the characteristics of the model. The comparative ROC for the indices calculated as described shows an improved potential for predicting POD with an AUCROC of 0.84 (0.72–0.94, *p* < 0.001) for the optimized index (Figure 3, Table 4). The sensitivity and specificity of the optimized model were 0.61 and 0.93, with an accuracy of 0.86.

**Table 3:** Indices and contributing parameters are given as medians and IQRs; *p*-values are given for two-group tests regarding the presence of POD results from *t*-tests or wilcox tests accordingly.

	POD+	POD −	*p*-value 2-group-test
CARDEL index	9.9 [9.19–10.17]	8.75 [8–9.49]	0.0026
CARDEL index adapted	15.57 [13.63–16.55]	12.37 [10.13–14.45]	0.0060
Age	71.5 [63.75–76.25]	64 [56–71]	0.0033
Preoperative HbA1c	6.55 [6.03–7.3]	5.85 [5.6–6.6]	0.0046
Preoperative PWR	26.67 [22.07–34.03]	31.72 [25.52–36.67]	0.0756
Extended CARDEL index	22.67 [19.66–25.28]	18.16 [15.96–19.86]	0.0002
Decrease in BChE activity (%)	31.82 [25.05–37.42]	23.82 [17.84–30.25]	0.0018
Index for optimized model	0.41 [−4.25–1.56]	−6 [−8.89–4.23]	2.8 × 10^−5^
Proportion Of Post OP Hb (%)	66.44 [61.82–75.74]	73.13 [69.82–80.85]	0.0029

The CARDEL index was calculated with beta coefficients given by kotfis et al., whereas for the adapted CARDEL index, beta coefficients inferred from our data set were used for the adapted CARDEL index. BChE, butyrylcholinesterase; POD, postoperative delirium; PWR, platelet-to-white-blood-cell ratio.

**Table 4:** Multivariate analysis—optimized model with AUC 0.84 [0.72–0.94] (*p* < 0.001).

Variables of the optimized model	beta coefficient	OR	*p-v*alue
age	0.11	1 [0.94–1.1]	0.55
preoperative HbA1c	0.29	2.5 [1.2–4.8]	0.01
decrease in BChE activity (%)	0.27	1.1 [1.0–1.2]	0.03
postoperative hemoglobin proportion (%)	−0.31	0.86 [0.78–0.96]	0.0045

AUC, area under the curve.

The multivariable model has been corrected for age [1 (0.94–1.1), *p* = 0.55] which is not an independent risk factor [AUC without age = 0.83 (0.72–0.93)].

## Discussion

This prospective study evaluated the CARDEL index in a homogenous cohort of patients undergoing elective CABG surgery. Despite the small study size of this derivation cohort and the lack of an external validation the study offers two main findings: First, it showed that the predictive power of the original and adapted CARDEL index for detecting POD is only moderate and can be optimized. In its original configuration, it showed only an AUCROC of 0.74. Second, multivariate regression analysis confirmed most parameters of the CARDEL index but also excluded the PWR as an independent risk factor for POD development. Instead, the inclusion of the drop in the BChE activity in an optimized model showed an increase in the predictive power of the score [AUCROC 0.84 (0.72–0.94)].

Our study's focus on a homogenous cohort of low-risk CABG patients, characterized by shorter cross-clamp times and standardized perioperative management, likely contributed to the relatively low POD incidence compared to studies including higher-risk patient groups such as those undergoing valvular surgery. As POD impacts the patient's outcome and quality of life, it is important to implement preventive strategies to avoid POD and to predict POD as accurately as possible to treat it early and effectively. Thus, the CARDEL index was designed only with parameters available prior to surgery. Even though this approach is useful, in our study, it only achieved sufficient prediction of POD, although the study collectives were comparable regarding the performed surgery and basic characteristics of the study collective (21). Moreover, calculating the CARDEL index resulted in a very similar prognostic power compared to our data (both 0.74). Recently, another study derived from the same study group evaluated the CARDEL index as a prognostic score but showed an AUCROC of only 0.664 for predicting POD (29). The incidence of POD was higher in our study [own results: 20.5%, Kotfis et al.: 13.3%, Lechowicz et al.: 14.9% (21, 29),], but most importantly, we were unable to confirm the PWR as the strongest risk factor for POD. This may be due to several reasons. First, the PWR represents a surrogate for the patient's general condition rather than their neuroinflammatory status and can vary between individuals. For example, in contrast to our study, Kotfis et al. also included patients with acute myocardial infarction, which can influence blood cell ratios due to the endothelial inflammatory response (21, 30–32). Moreover, several other factors could have influenced the PWR in the study collectives, such as other pre-existing diseases or ICU treatment. Second, standardization of PWR measurements is lacking. This affects not only the analysis techniques but also reference ranges. Since both studies investigating the CARDEL index originate from the same department, their analytics will be identical; however, they may differ from our laboratory. Finally, whether PWR or other ratios such as platelet-to-lymphocyte or platelet-to-neutrophil should be used in the context of neuroinflammation remains widely discussed because lymphocytes and neutrophils might be more specific for an inflammatory response (33, 34). For example, a recent meta-analysis of 11,579 critically ill patients did not include the PWR but only the platelet-to-lymphocyte and platelet-to-neutrophil ratios (35). In this analysis, the platelet-to-neutrophil ratio was prognostic for delirium, while the platelet-to-lymphocyte ratio failed. Overall, blood cell ratios might contribute to detecting POD but are probably insufficiently specific for single use as a predictive biomarker.

Based on our previous study showing that a drop in BChE activity correlated well with the onset of POD, we assumed that this might improve the prediction of the CARDEL index and demonstrate an increase in the AUC to 0.80. Finally, we reviewed our data with regression analysis and identified elevated HbA1c, a decrease in BChE activity, and a postoperative change in hemoglobin as risk factors for POD development, which we included in our prediction model and achieved an AUC of 0.84 with a sensitivity and specificity of 0.61 and 0.93, respectively. It is well-documented that anemia during cardiac surgery is associated with POD development (7, 36, 37). For example, the IPDACS study showed that the risk for POD increased more than fourfold when patients suffered from anemia (7). Furthermore, diabetes also presents a known risk factor for POD, although it is not yet determined whether diabetes or hyperglycemia contribute most to POD development (38–40). Thus, it was unsurprising that anemia and diabetes were also identified as risk factors for POD in our study. However, the risk of developing POD in diabetic patients (quantified with HbA1c) was similar to findings from a study by Kotfis et al. (*n* = 3,178), which reported an odds ratio of 1.269 (95% CI: 1.161–1.387, *p* < 0.001) (41). In contrast, a large cohort study of 25,532 Korean cardiac surgical patients revealed that acute hyperglycemia, but not chronic hyperglycemia, increased the risk for POD (39). The underlying reasons for these controversial findings are still not fully understood. While acute hyperglycemia may cause neuroinflammation, making patients more vulnerable to POD, those with chronic hyperglycemia might have developed protective cellular responses (42).

This is particularly relevant because all parameters were available immediately after surgery, enabling early and accurate prediction of POD, which may compensate for the limitation of not having all parameters available preoperatively. BChE might present a promising target as a prognostic biomarker for POD. Alongside other studies, the underlying observational study of this secondary analysis showed alterations in the BChE activity that were predictive of POD in cardiac surgical patients (17–19). Conversely, a recent study by Schlake et al. did not show a relationship between the BChE activity and POD after cardiac surgery, although this activity decreased significantly postoperatively (20). A possible explanation might lie in the differences between the included patients. While we included only patients undergoing CABG, Schlake et al. included various types of cardiac surgical patients with and without the use of CPB. Because CPB leads to a strong inflammatory response, it significantly influences the onset of POD and might also impact the drop in BChE activity. Although no difference between patients with and without CPB was identified regarding the onset of POD, this phenomenon is well-known (4, 22). Further, the cause of BChE drop must be discussed. Since cardiac surgery can influence the liver function and, therefore, also the synthesis of BChE, dynamics of liver enzymes must be taken in account. In this study, liver enzymes did not differ between patients with and without patients and stayed overall in a normal range. For this reason, it is unlikely that disturbances of the liver function influenced the BChE activity. Another possible influencing factor on BChE activity are RBC. Even though BChE is mainly found in the plasma and acetylcholinesterase in red blood cells, the activity of BChE is also strongly correlated to the amount of the red blood cell count (28). Even though BChE is primarily found in the plasma and acetylcholinesterase in red blood cells, the activity of BChE is also strongly correlated to the amount of the RBC count. It is therefore possible that RBC transfusion might influence the BChE activity. In fact, the amount of RBC transfusion was higher in patients with POD raising the question how this finding might interact with the significant decrease of BChE activity in patients with POD. Since it is known that RBC transfusions modulate inflammatory reactions, and systemic inflammation is associated with a decrease in BChE activity (43–46), there may be a connection between RBC transfusions and the development of POD, as has been described in previous studies (47–49). Finally, it is notable that Schlake et al. did not measure the drop in BChE activity in the first two days after surgery, while our study only included measurements of the very early stages of the BChE activity (immediately after surgery and at day 1). Therefore, the BChE activity might already have been regenerated in the study by Schlake et al., which would explain the statistically non-significant differences. Moreover, two other studies did not show a prognostic value of BChE; however, the study designs differed from our study. While John et al. did not include preoperative values and therefore could not show a perioperative drop in BChE activity (18), Michels et al. used a composite endpoint (POD, acute renal failure, pneumonia, and arrhythmia) (50). Overall, these contradicting results underline the need for studies with larger numbers of patients and clearly defined target parameters (e.g., drop in BChE activity).

This study features some important limitations. First, due to its design as a secondary analysis, we could not increase the number of included patients. Therefore, no sample size calculations were performed. This led to a small sample size in the derivation cohort, which needs to be reevaluated through external validation. Second, although we attempted to reduce influencing factors, it remains possible that chronic health conditions might have influenced the BChE activity. For example, cholinesterase deficiency syndromes, genetic variants, polymorphisms and mutations in BChE activity represent a potential confounder in studies on perioperative cholinergic disturbance (51). Future studies should include an assessment of frailty to describe the patients' status more thoroughly. Next, although all surgeries were performed during the daytime, the circadian rhythm might still have influenced BChE plasma levels. Third, the anticholinergic effects of the given drugs cannot be excluded; however, these patients underwent a highly standardized surgery and anesthesia, making them comparable with a simultaneously very low drug-related anticholinergic burden (52). Although the exclusion criteria (e.g., autoimmune diseases, severe psychiatric disorders) and anesthetic management were designed to minimize the risk of exposure to such medications, it cannot be ruled out that some drugs may have still influenced BChE activity. For instance, five patients were treated with antidepressant medications, which may have influenced BChE levels. Due to the secondary analysis design of the study, this limitation could not be avoided. Finally, in 14 of the POD-patients and two of the POD+ group, the CARDEL index was not calculable due to missing HbA1c values.

## Conclusion

In summary, this study identified an elevated HbA1c as the strongest risk factor for the development of POD followed by the decrease in BChE activity and postoperative anemia, respectively age. Furthermore, the predictive power of the CARDEL index was significantly improved by including these factors. The PWR was not associated with the onset of POD after cardiac surgery, while point-of-care measurement of BChE activity enhanced the predictive quality of the CARDEL index. All parameters of the optimized prognostic model are easily available within the first 24 h after surgery, making the score potentially useful for early risk assessment. It could support conversations with patients and families by providing an early, individualized risk assessment for POD in the postoperative period. However, it must be highlighted that this study is based on a small derivation cohort and did not yet undergo external validation.

## Data Availability

The original contributions presented in the study are included in the article/supplementary material, further inquiries can be directed to the corresponding author.

## References

[1] Aldecoa C Bettelli G Bilotta F Sanders RD Audisio R Borozdina A European Society of Anaesthesiology evidence-based and consensus-based guideline on postoperative delirium. Eur J Anaesthesiol. (2017) 34(4):192–214. 10.1097/EJA.0000000000000594 28187050

[2] Jin Z Hu J Ma D. Postoperative delirium: perioperative assessment, risk reduction, and management. Br J Anaesth. (2020) 125(4):492–504. 10.1016/j.bja.2020.06.063 32798069

[3] Sanson G Khlopenyuk Y Milocco S Sartori M Dreas L Fabiani A. Delirium after cardiac surgery. Incidence, phenotypes, predisposing and precipitating risk factors, and effects. Heart Lung. (2018) 47(4):408–17. 10.1016/j.hrtlng.2018.04.005 29751986

[4] O’Neal JB Shaw AD. Predicting, preventing, and identifying delirium after cardiac surgery. Perioper Med. (2016) 5(1):7. 10.1186/s13741-016-0032-5 27119013 PMC4845390

[5] Salluh JIF Wang H Schneider EB Nagaraja N Yenokyan G Damluji A Outcome of delirium in critically ill patients: systematic review and meta-analysis. Br Med J. (2015) 350(3):h2538. 10.1136/bmj.h2538 26041151 PMC4454920

[6] Smulter N Lingehall HC Gustafson Y Olofsson B Engström KG. Delirium after cardiac surgery: incidence and risk factors. Interact Cardiovasc Thorac Surg. (2013) 17(5):790–6. 10.1093/icvts/ivt323 23887126 PMC3805209

[7] Kazmierski J Kowman M Banach M Fendler W Okonski P Banys A Incidence and predictors of delirium after cardiac surgery: results from the IPDACS study. J Psychosom Res. (2010) 69(2):179–85. 10.1016/j.jpsychores.2010.02.009 20624517

[8] Gosselt ANC Slooter AJC Boere PRQ Zaal IJ. Risk factors for delirium after on-pump cardiac surgery: a systematic review. Crit Care. (2015) 19(1):1–8. 10.1186/s13054-014-0721-8 26395253 PMC4579578

[9] Darvesh S Hopkins DA Geula C. Neurobiology of butyrylcholinesterase. Nat Rev Neurosci. (2003) 4(2):131–8. 10.1038/nrn1035 12563284

[10] Maldonado JR. Delirium pathophysiology: an updated hypothesis of the etiology of acute brain failure. Int J Geriatr Psychiatry. (2018) 33(11):1428–57. 10.1002/gps.4823 29278283

[11] Sun T Zhen T Harakandi CH Wang L Guo H Chen Y New insights into butyrylcholinesterase: pharmaceutical applications, selective inhibitors and multitarget-directed ligands. Eur J Med Chem. (2024) 275:116569. 10.1016/j.ejmech.2024.116569 38852337

[12] Lockridge O. Review of human butyrylcholinesterase structure, function, genetic variants, history of use in the clinic, and potential therapeutic uses. Pharmacol Ther. (2015) 148:34–46. 10.1016/j.pharmthera.2014.11.011 25448037

[13] Jahangir S Allala M Khan AS Muyolema Arce VE Patel A Soni K A review of biomarkers in delirium superimposed on dementia (DSD) and their clinical application to personalized treatment and management. Cureus. (2023) 15(5):e38627. 10.7759/cureus.38627 37159618 PMC10163832

[14] Zhao B Ni Y Tian X. Low plasma cholinesterase activity is associated with postoperative delirium after noncardiac surgery in elderly patients: aProspective observational study. Psychosomatics. (2019) 60(2):190–196. 10.1016/j.psym.2018.06.014 30093245

[15] Müller A Olbert M Heymann A Zahn PK Plaschke K Von Dossow V Relevance of peripheral cholinesterase activity on postoperative delirium in adult surgical patients (CESARO): a prospective observational cohort study. Eur J Anaesthesiol. (2019) 36(2):114–122. 10.1097/EJA.0000000000000888 30431498

[16] Gruendel MS Brenneisen W Wollborn J Haaker G Meersch M Gurlit S Perioperative point-of-care-testing of plasmacholinesterases identifies older patients at risk for postoperative delirium: an observational prospective cohort study. BMC Geriatr. (2024) 24(1):136. 10.1186/s12877-023-04627-1 38321383 PMC10848373

[17] Zajonz TS Kunzemann C Schreiner AL Beckert F Schneck E Boening A Potentials of acetylcholinesterase and butyrylcholinesterase alterations in on-pump coronary artery bypass surgery in postoperative delirium: an observational trial. J Clin Med. (2023) 12(16):5245. 10.3390/jcm12165245 37629287 PMC10455192

[18] John M Ely EW Halfkann D Schoen J Sedemund-Adib B Klotz S Acetylcholinesterase and butyrylcholinesterase in cardiosurgical patients with postoperative delirium. J Intensive Care. (2017) 5(1):29. 10.1186/s40560-017-0224-1 28560042 PMC5446746

[19] Adam EH Haas V Lindau S Zacharowski K Scheller B. Cholinesterase alterations in delirium after cardiosurgery: a German monocentric prospective study. BMJ Open. (2020) 10(1):e031212. 10.1136/bmjopen-2019-031212 31941763 PMC7044931

[20] Schlake K Teller J Hinken L Laser H Lichtinghagen R Schäfer A Butyrylcholinesterase activity in patients with postoperative delirium after cardiothoracic surgery or percutaneous valve replacement- an observational interdisciplinary cohort study. BMC Neurol. (2024) 24(1):80. 10.1186/s12883-024-03580-9 38424490 PMC10905803

[21] Kotfis K Ślozowska J Safranow K Szylińska A Listewnik M. The practical use of white cell inflammatory biomarkers in prediction of postoperative delirium after cardiac surgery. Brain Sci. (2019) 9(11):308. 10.3390/brainsci9110308 31684066 PMC6896029

[22] Lopez MG Hughes CG DeMatteo A O’Neal JB McNeil JB Shotwell MS Intraoperative oxidative damage and delirium after cardiac surgery. Anesthesiology. (2020) 132(3):551–61. 10.1097/ALN.0000000000003016 31770146 PMC7015795

[23] Lindroth H Bratzke L Purvis S Brown R Coburn M Mrkobrada M Systematic review of prediction models for delirium in the older adult inpatient. BMJ Open. (2018) 8(4):e019223. 10.1136/bmjopen-2017-019223 29705752 PMC5931306

[24] World Medical Association. World medical association declaration of Helsinki. JAMA. (2013) 310(20):2191. 10.1001/jama.2013.281053 24141714

[25] von Elm E Altman DG Egger M Pocock SJ Gøtzsche PC Vandenbroucke JP. The strengthening the reporting of observational studies in epidemiology (STROBE) statement: guidelines for reporting observational studies. J Clin Epidemiol. (2008) 61(4):344–9. 10.1016/j.jclinepi.2007.11.008 18313558

[26] Worek F Mast U Kiderlen D Diepold C Eyer P. Improved determination of acetylcholinesterase activity in human whole blood. Clin Chim Acta. (1999) 288(1–2):73–90. 10.1016/S0009-8981(99)00144-8 10529460

[27] Securetec Detection-Systems AG. ChE check mobile: portable cholinesterase testing system. Neubibberg: Securetec Detection-Systems AG (2013). Available online at: https://www.securetec.net/app/uploads/2018/08/Schnelltest-Bestimmung-Cholinesterase_Brochure_ChE_Enzymtest_military_use_70507_v03_EN_Email.pdf (accessed December 02, 2024).

[28] Jasiecki J Targońska M Wasąg B. The role of butyrylcholinesterase and iron in the regulation of cholinergic network and cognitive dysfunction in Alzheimer’s disease pathogenesis. Int J Mol Sci. (2021) 22(4):2033. 10.3390/ijms22042033 33670778 PMC7922581

[29] Lechowicz K Szylińska A Listewnik M Drożdżal S Tomska N Rotter I Cardiac delirium index for predicting the occurrence of postoperative delirium in adult patients after coronary artery bypass grafting. Clin Interv Aging. (2021) 16:487–95. 10.2147/CIA.S302526 33762820 PMC7982438

[30] Wang H Li L Ma Y. Platelet-to-lymphocyte ratio a potential prognosticator in acute myocardial infarction: a prospective longitudinal study. Clin Cardiol. (2023) 46(6):632–8. 10.1002/clc.24002 37060180 PMC10270260

[31] Dong G Huang A Liu L. Platelet-to-lymphocyte ratio and prognosis in STEMI: a meta-analysis. Eur J Clin Invest. (2021) 51(3):e13386. 10.1111/eci.13386 32810283

[32] Chen Y Chen S Han Y Xu Q Zhao X. Neutrophil-to-lymphocyte ratio and platelet-to-lymphocyte ratio are important indicators for predicting in-hospital death in elderly AMI patients. J Inflamm Res. (2023) 16:2051–61. 10.2147/JIR.S411086 37215380 PMC10198281

[33] Islam MM Satici MO Eroglu SE. Unraveling the clinical significance and prognostic value of the neutrophil-to-lymphocyte ratio, platelet-to-lymphocyte ratio, systemic immune-inflammation index, systemic inflammation response index, and delta neutrophil index: an extensive literature re. Turk J Emerg Med. (2024) 24(1):8–19. 10.4103/tjem.tjem_198_23 38343523 PMC10852137

[34] Buonacera A Stancanelli B Colaci M Malatino L. Neutrophil to lymphocyte ratio: an emerging marker of the relationships between the immune system and diseases. Int J Mol Sci. (2022) 23(7):3636. 10.3390/ijms23073636 35408994 PMC8998851

[35] Sarejloo S Shojaei N Lucke-Wold B Zelmanovich R Khanzadeh S. Neutrophil to lymphocyte ratio and platelet to lymphocyte ratio as prognostic predictors for delirium in critically ill patients: a systematic review and meta-analysis. BMC Anesthesiol. (2023) 23(1):58. 10.1186/s12871-023-01997-2 36803215 PMC9942068

[36] Kulier A Levin J Moser R Rumpold-Seitlinger G Tudor IC Snyder-Ramos SA Impact of preoperative anemia on outcome in patients undergoing coronary artery bypass graft surgery. Circulation. (2007) 116(5):471–9. 10.1161/CIRCULATIONAHA.106.653501 17620512

[37] Li MM Miles S Callum J Lin Y Karkouti K Bartoszko J. Postoperative anemia in cardiac surgery patients: a narrative review. Can J Anaesth. (2023) 71(3):408–421. 10.1007/s12630-023-02650-9 38017198

[38] Li J Meng D Chang C Fu B Xie C Wu Z Risk factors for delirium after coronary artery bypass grafting in elderly patients. Ann Transl Med. (2021) 9(22):1666. 10.21037/atm-21-5160 34988175 PMC8667104

[39] Park SJ Oh AR Lee J-H Yang K Park J. Association of preoperative blood glucose level with delirium after non-cardiac surgery in diabetic patients. Korean J Anesthesiol. (2024) 77(2):226–235. 10.4097/kja.23301 38171594 PMC10982528

[40] Oh AR Lee DY Lee S Lee J-H Yang K Choi B Association between preoperative glucose dysregulation and delirium after non-cardiac surgery. J Clin Med. (2024) 13(4):932. 10.3390/jcm13040932 38398245 PMC10889204

[41] Kotfis K Szylińska A Listewnik M Brykczyński M Ely EW Rotter I. Diabetes and elevated preoperative hba1c level as risk factors for postoperative delirium after cardiac surgery: an observational cohort study. Neuropsychiatr Dis Treat. (2019) 15:511–521. 10.2147/NDT.S196973 30863073 PMC6388975

[42] Marik PE Bellomo R. Stress hyperglycemia: an essential survival response!. Crit Care. (2013) 17(2):305. 10.1186/cc12514 23470218 PMC3672537

[43] Remy KE Hall MW Cholette J Juffermans NP Nicol K Doctor A Mechanisms of red blood cell transfusion-related immunomodulation. Transfusion (Paris). (2018) 58(3):804–815. 10.1111/trf.14488 PMC6592041 29383722

[44] Zivkovic AR Schmidt K Sigl A Decker SO Brenner T Hofer S. Reduced serum butyrylcholinesterase activity indicates severe systemic inflammation in critically ill patients. Mediators Inflamm. (2015) 2015:274607. 10.1155/2015/274607 25762852 PMC4339712

[45] Lampón N Hermida-Cadahia EF Riveiro A Tutor JC. Association between butyrylcholinesterase activity and low-grade systemic inflammation. Ann Hepatol. (2012) 11(3):356–363. 10.1016/S1665-2681(19)30932-9 22481455

[46] Hod EA Zhang N Sokol SA Wojczyk BS Francis RO Ansaldi D Transfusion of red blood cells after prolonged storage produces harmful effects that are mediated by iron and inflammation. Blood. (2010) 115(21):4284–4292. 10.1182/blood-2009-10-245001 20299509 PMC2879099

[47] Mariscalco G Biancari F Juvonen T Zanobini M Cottini M Banach M Red blood cell transfusion is a determinant of neurological complications after cardiac surgery. Interact Cardiovasc Thorac Surg. (2015) 20(2):166–171. 10.1093/icvts/ivu360 25368133

[48] Brown CH Grega M Selnes OA McKhann GM Shah AS Laflam A Length of red cell unit storage and risk for delirium after cardiac surgery. Anesth Analg. (2014) 119(2):242–250. 10.1213/ANE.0000000000000134 24859077 PMC4107069

[49] Lee S-S Kim J-H Lee J-J Kwon Y-S Seo E-M. The impact of blood transfusion in developing postoperative delirium in patients with hip fracture surgery. J Clin Med. (2023) 12(14):4696. 10.3390/jcm12144696 37510810 PMC10380490

[50] Michels B Holzamer A Graf BM Bredthauer A Petermichl W Müller A Butyrylcholinesterase as a perioperative complication marker in patients after transcatheter aortic valve implantation: a prospective observational study. BMJ Open. (2021) 11(7):e042857. 10.1136/bmjopen-2020-042857 PMC8261881 34230011

[51] Lane RM He Y. Butyrylcholinesterase genotype and gender influence Alzheimer’s disease phenotype. Alzheimers Dement. (2013) 9(2):e1–73. 10.1016/j.jalz.2010.12.005 22402324

[52] Hilmer SN Gnjidic D. The anticholinergic burden: from research to practice. Aust Prescr. (2022) 45(4):118–20. 10.18773/austprescr.2022.031 36110165 PMC9427617

